# The effect of estradiol on granulosa cell responses to FSH in women with polycystic ovary syndrome

**DOI:** 10.1186/s12958-017-0230-0

**Published:** 2017-02-10

**Authors:** Michael V. Homer, Marcus A. Rosencrantz, Rana F. Shayya, R. Jeffrey Chang

**Affiliations:** 1 Reproductive Medicine, University of California, 200 West Arbor Drive MC: 8710, San Diego, CA 92103 USA; 2 Reproductive Endocrinology and Infertility, Southern California Permanente Medical Group, 6650 Alton Parkway, Irvine, CA 92618 USA; 3 Obstetrics and Gynecology, Southern California Permanente Medical Group, 250 Travelodge Drive, El Cajon, CA 92020 USA; 4 9500 Gilman Drive #0633 La Jolla, San Diego, CA 92093 USA

**Keywords:** Polycystic ovary syndrome, Granulosa cell, Estradiol, FSH, Inhibin B

## Abstract

**Background:**

The influence of estradiol (E_2_) on granulosa cell (GC) function has not been tested clinically in women with polycystic ovary syndrome (PCOS). The objective of this study is to determine if E_2_ influences GC responses to FSH in women with PCOS.

**Methods:**

This is a two phase, single cohort study conducted over a 2-year period at a single academic center. Nine women with PCOS according to NIH criteria. In Phase 1, FSH stimulation of GC responses as measured by E_2_ and Inhibin B (Inh B) were assessed before and at 5 and 6 weeks after GnRH agonist administration. In Phase 2, the same protocol was employed with the addition of an aromatase inhibitor (letrozole, LET) administered daily beginning at week 4 for 2 weeks.

**Results:**

In Phase 1, recovery of FSH, E_2_ and Inh B from ovarian suppression occurred at 5 and 6 weeks after GnRH agonist injection and preceded resumption of LH and androgen secretion. In Phase 2, hormone recovery after GnRH agonist was characterized by elevated FSH and suppressed E_2_ levels whereas recovery of LH and androgen levels were unchanged. In Phase 1, FSH stimulated E_2_ and Inh B responses were unaltered during recovery from ovarian suppression. In Phase 2, E_2_ and Inh B fold changes after FSH were significantly reduced at weeks 5 (*p <* 0.04) and 6 (*p <* 0.01), respectively.

**Conclusion:**

In anovulatory women with PCOS, chronic, unopposed E_2_ secretion may contribute, at least in part, to enhanced ovarian responsiveness to FSH.

**Trial registration:**

NCT02389088

## Background

The characteristic features of polycystic ovary syndrome (PCOS) are anovulation, androgen excess, and polycystic ovary morphology. Anovulation is associated with modest estradiol (E_2_) secretion derived primarily from peripheral extraglandular conversion and minimal progesterone production. However, in vitro and in vivo studies have demonstrated that granulosa cells (GCs) from women with PCOS are hyperresponsive to FSH stimulation compared to responses observed in normal GCs. These findings suggest that the ovary may contribute to circulating E_2_ in women with this disorder [1–3].

Local production of E_2_ may account, at least in part, for the enhanced CG response to FSH in PCOS. There is considerable evidence that estrogen enhances follicle function including responses to FSH. Diethylstilbestrol treated GCs from immature hypophysectomized rats produced greater E_2_ release in response to FSH compared to that of untreated cells [4]. In addition, estrogen has been shown to influence GC cytodifferentiation by modulating follicular intracellular gap junctions, estrogen receptor content, and adenylate cyclase activity [5–7]. Synergy between E_2_ and FSH has been demonstrated in GCs with regards to increased FSH receptor binding and increased aromatase activity [8–10]. Corresponding in vivo efforts to demonstrate an effect of estrogens on GC E_2_ production in women has been understandably difficult and not undertaken.

Previously, we and others have shown that in women with PCOS serum E_2_ and inhibin B (Inh B) exhibit similar temporal responsiveness to FSH [11, 12]. These findings suggest that Inh B may serve as a marker of granulosa cell activity alongside E_2_. In an effort to determine whether estrogen influences GC function in women with PCOS, we examined Inh B responses to FSH in the presence or absence of an aromatase inhibitor.

## Methods

### Participants

Nine women with PCOS were recruited for study. All subjects were between 18 and 35 years of age and exhibited clinical and laboratory evidence of hyperandrogenism, were either oligomenorrheic or amenorrheic and had greater than 12 antral follicles per ovary on transvaginal ultrasound that are consistent with criteria established by the NIH, Rotterdam, and Androgen Excess-PCOS Society. Mean age (± SE) was 26 ± 1.2 years and BMI (± SE) was 33.7 ± 2.4 kg/m^2^. Serum levels of 17-hydroxyprogesterone (17-OHP), TSH, and prolactin were within normal range. All patients were free of medication containing hormones for 3 months or greater prior to study.

The study was approved by the Human Research Protection Program at the University of California, San Diego (UCSD, IRB#100023) and written informed consent was obtained from each participant before study.

### Procedures

The study was divided into two phases. For Phase I of the study, participants were admitted on a random day to the Clinical and Translational Research Institute (CTRI) at UC San Diego for testing. Patients all had serum progesterone levels < 1.5 ng/ml and negative hCG tests. After placement of an intravenous line, each subject received an intravenous injection of recombinant human FSH (r-hFSH), 150 IU. Blood samples were obtained prior to and 24 h afterwards. After the last blood sample was obtained, each was given long-acting GnRH agonist (Depot Lupron), 3.75 mg intramuscularly, to maximally suppress ovarian steroid production for one month after which serum E_2_ levels gradually resume [13]. Ovarian suppression was employed to allow for FSH stimulation studies to be conducted during the recovery phase when the ovary and GCs regain responsiveness, thereby establishing uniform and comparable E_2_ levels in both phases of study. During this interval, FSH stimulation was repeated at the end of week 5 and week 6.

For Phase II of the study, the same 9 participants were allowed to have a hormone-free washout period of 3 months from administration of GnRH agonist and 2 months from the prior dose of FSH. They were then re-admitted to the CTRI at UC San Diego for study. Similar to Phase I, they underwent a baseline FSH stimulation test after which 3.75 mg of Depot Lupron was administered. At the beginning of week 4, each subject received an aromatase inhibitor, Letrozole (LET), 5 mg daily, for 2 weeks to restrict increases of serum E_2_ levels that were observed in Phase I. The FSH stimulation test was then repeated at the end of week 5 and week 6.

### Assays

Serum concentrations of LH and FSH were measured by radio-immunoassay (RIA) with intra— and inter-assay coefficients of variation (CV) of 5.4 and 8.0%, respectively, for LH and 3.0 and 4.6%, respectively, for FSH (Diagnostic Products Corp., Los Angeles, CA, USA). Serum concentrations of Inh B were measured by ELISA with inter— and intra-assay CV of 6.7 and 4.6% (Diagnostic Systems Laboratories, Inc., Webster, TX, USA). The highly specific two-site ELISA Kit allows for quantitative measurement of dimeric Inh B in human serum. Assay sensitivity for Inh B was 7.0 pg/ml. Serum concentrations of E_2_, androstenedione (A) and testosterone (T) were measured by well-established RIA with intra-assay CV less than 7%. P_4_, 17-OHP and dehydroepiandrosterone sulfate (DHEAS) were measured by RIA with intra-assay CV less than 7% (Diagnostic Systems Laboratories, Inc., Webster, TX, USA).

### Statistical analysis

Q-Q plot and boxplot were used to check the normality of the data. One-sample t-test and one-sample Wilcoxon signed-rank test were applied as needed to test the differences of hormone measurements at both baseline and after FSH stimulation for Phase I compared to Phase II. We also tested for differences in the log fold change of E_2_, Inh B and insulin hormone before and after FSH. For all analysis, p values of < 0.05 were considered statistically significant. Statistical analysis were performed using the R statistical computing software (version 2.6.2, http://www.r-project.org, 2009).

## Results

### Serum hormone levels before and after GnRH agonist ovarian suppression (phase I)

**Fig. 1:**
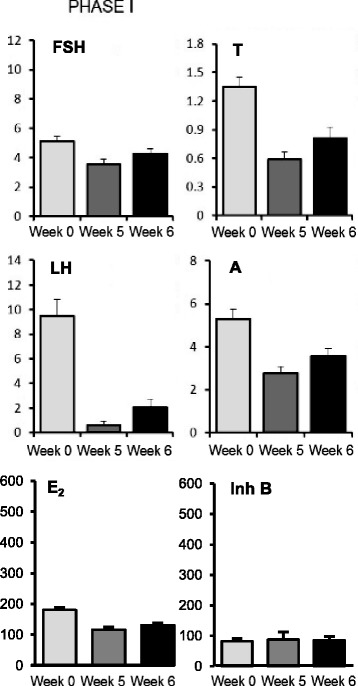
Serum hormone levels before and during recovery from GnRH agonist. Serum levels of FSH (IU/L), LH (IU/L) T (nmol/L), A (nmol/L), E_2_ (pmol/L) and Inh B (ng/L)

Baseline circulating hormone levels for women with PCOS are shown in Table 1. Mean circulating levels of LH, FSH, A, T, E_2_ and Inh B before and following pituitary gonadotropin desensitization and ovarian suppression are shown in Fig. 1. As expected, serum E_2_ levels at the end of week 5 and week 6 after GnRH agonist approximated pretreatment levels that reflected recovery from ovarian suppression. Basal Inh B levels at the end of 5 and 6 weeks after GnRH agonist were not different compared to baseline serum values. Serum FSH levels at the end of week 5 were similar to baseline concentrations indicating complete recovery from pituitary desensitization. By comparison, LH and serum androgens at the end of week 5 remained lower than pre-GnRH agonist treatment levels. Subsequently, there were subtle increases of LH and androgen production by the end of week 6.

**Table 1:** Baseline circulating steroid hormone and gonadotropin levels in women with PCOS.

	PCOS (*n =* 9)
LH (IU/L)	9.01 [5.95 – 12.87]
FSH (IU/L)	4.98 [4.36 – 5.48]
T (nmol/L)	1.3 [1.17 – 1.54]
A (nmol/L)	4.84 [4.55 – 6.52]
17OH-P (nmol/L)	46.36 [34.85 – 49.39]
E_2_ (pmol/L)	179 [158–204]

Data are expressed as Median [IQ Range]). To convert to gravimetric units, multiply by the following conversion factors: T [ng/dL], 28.82; A [ng/dL], 28.65; 17OH-P [ng/dL], 3.30; E_2_ [pg/mL], 0.27

### Serum hormone levels before and after GnRH agonist ovarian suppression in the presence of LET (phase II)

**Fig. 2:**
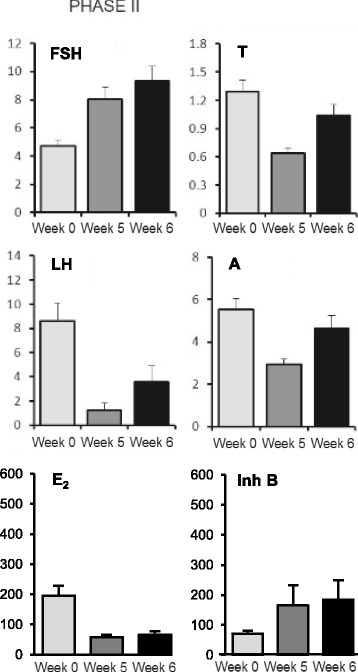
Serum hormone levels before and during recovery from GnRH agonist together with LET. Serum levels of FSH (IU/L), LH (IU/L) T (nmol/L), A (nmol/L), E_2_ (pmol/L) and Inh B (ng/L)

In Phase II, corresponding levels of gonadotropins, E_2_, Inh B and androgens associated with the administration of LET during weeks 4 and 5 after GnRH agonist injection are shown in Fig. 2. Basal E_2_ levels were significantly lower during daily LET administration (weeks 5 and 6) compared to corresponding values observed in Phase 1. Accordingly, these reduced E_2_ levels were associated with significantly higher basal serum FSH levels at week 5 and 6 (*p <* 0.01) as shown in Fig. 2. Notably, these increments of FSH were significantly greater than those observed without LET. In contrast to E_2_, basal serum Inh B levels exhibited increases during LET treatment compared to the mean baseline value at week 0, although these increments were not statistically significant. In Phase 2 patterns of increasing basal values of LH, A and T levels during recovery were similar to those observed in Phase 1.

### E_2_ and Inh B responses to FSH during recovery from GnRH suppression in the absence and presence of LET

In Phase I, the FSH-stimulated fold-changes of E_2_ at 5 and 6 weeks during recovery were not significantly different from that observed at baseline, prior to GnRH agonist administration (Table 2). However, in Phase II studies with LET treatment, reduced basal E_2_ levels were accompanied by a corresponding decline of the fold-change E_2_ responses to FSH injection at weeks 5 (*p <* 0.04) and 6 (*p <* 0.01) compared to baseline. Similarly, Inh B responses to FSH in were not different in Phase I, but significantly decreased fold-changes following FSH were observed at 5 (*p <* 0.04) and 6 (*p <* 0.01) weeks.

**Table 2:** Serum estradiol and Inh B fold-change responses to FSH at week 0 (baseline), week 5 and week 6 during Phase I (without LET) and Phase II (with LET) of study

	Week 0	Week 5	Week 6
Estradiol			
Phase I	2.53 ± 0.25	2.14 ± 0.24	2.70 ± 0.27
Phase II	2.20 ± 0.18	1.39 ± 0.16^a^	1.63 ± 0.20^b^
Inh B			
Phase I	5.31 ± 0.55	5.82 ± 0.88	6.08 ± 0.78
Phase II	5.50 ± 0.25	3.33 ± 0.48^a^	3.14 ± 0.43^b^

^a^ *p <* 0.04, significantly different from Week 0

^b^ *p <* 0.01, significantly different from Week 0

## Discussion

The results of this study are consistent with a facilitative role for E_2_ on GC function in women with PCOS. This is suggested by the reduced fold-change responses of Inh B as well as E_2_ following FSH administration in the presence of lowered serum E_2_ levels during LET administration. Decreased responsiveness of Inh B was observed despite corresponding increases of FSH, which is known to enhance Inh B release.

Hyperresponsiveness to FSH administration in women with PCOS is a well-recognized phenomenon that predisposes to hyperstimulation during ovulation induction. This is likely due to the abundance of small antral follicles that exist in women with PCOS as well as an increased number of FSH receptors per granulosa cell in follicles from anovulatory women with this disorder [14, 15]. However, there is a lack of clinical studies in women regarding the role of E_2_ on GC function and follicle development. As a result, most of our understanding of how E_2_ impacts follicle health has been inferred from experiments in animal models. It is apparent that E_2_ does not exert a direct effect on GCs as E_2_ response elements have not been identified on the CYP19 promoter in the rat model or human GCs [16]. Rather, it is likely synergism between E_2_ and FSH that promotes GC function and follicle growth. For instance, studies have shown that maximal FSH stimulation of aromatase activation, antrum formation, and LH responsiveness in GCs requires E_2_ [4, 16–20]. These findings are underscored by studies conducted in β-estrogen receptor knock out mice that demonstrate the necessity of E_2_ to achieve maximum FSH action in GCs [21].

There were modest increases of basal serum Inh B during LET treatment in Phase II that were not statistically significant compared to Phase I. The subtle change of Inh B may have been induced by raised levels of circulating FSH as a result of lowered serum E_2_ levels rather than an inherent increase of GC responsiveness. Nevertheless, corresponding Inh B responses to FSH were not greater than those observed prior to GnRH agonist administration or Inh B responses in Phase I. Thus, the incremental fold-change was significantly less in Phase II in association with diminished E_2_ levels. Considering that Inh B may serve as a GC marker, this provides indirect evidence that estrogen augments E_2_ responsiveness to FSH in women with PCOS.

While these results suggest that E_2_ may contribute to enhanced GC function in women with PCOS, it is less clear as to whether chronic E_2_ secretion directly impacts GC hyper-responsiveness to FSH as demonstrated in vitro or in women with this disorder. In the hypogonadotropic hypogonadal female, serum E_2_ levels are low and initial ovarian responses to exogenous FSH are decreased compared to anovulatory women with normal E_2_ concentrations. Estradiol has also been used in poor ovarian responders to help increase the number of mature follicles retrieved and decrease cycle cancellations [22]. By comparison, anovulatory women with PCOS are distinctive in that efforts to induce ovulation appear to assume varying and, at times, apparent diametrical ovarian responses. In PCOS women initiation of ovulation induction has been characterized by a lack of follicle response that commonly warrants higher therapeutic doses or more intensive treatment modalities. It has been reported that during clomiphene citrate administration significantly smaller increments of E_2_ were observed compared to those of normal women despite comparable increases of serum FSH [23] As a result, several modified treatment regimens have been proposed that markedly increase the total dose of clomiphene or utilize additional drugs, including gonadotropins, to enhance clomiphene effectiveness. Primary gonadotropin therapy in women with PCOS has also been associated with poor initial ovarian responses to ovulation induction as early E_2_ responses to daily gonadotropin stimulation were considerably less compared to that found in normal women [24]. The mechanism of ovarian insensitivity in the early stages of ovulation induction has not been examined. In women with PCOS, chronic E_2_ secretion has been attributed to peripheral extra-glandular conversion and local intra-ovarian production may be insufficient to support early follicular responses to FSH. Alternatively, multiple factors, both intra- and extra-ovarian, may be responsible for or contribute to anovulation in women with this disorder and warrant further investigation. This would include AMH’s possible role in regulation of ovarian function and morphology [25, 26].

Despite the reduction of E_2_ during LET treatment, circulating levels of T and A were inexplicably not altered. Whether this was the result of LET superimposed on already suppressed ovarian steroidogenesis is uncertain, although it would seem that aromatase inhibition at extra-glandular sites might result in elevated serum androgens. Previously, it was reported that T as well as gonadotropins were unaltered in premenopausal women administered LET, 2.5 mg, daily over 12 weeks [27]. Another consideration may be that our study subjects were comprised of only those with PCOS with an elevated BMI and the results should not be generalized to a non-obese population due to BMI’s influence on insulin resistance and ovarian hormones.

The experimental design of our study was complex, but necessary in an attempt to fully assess the effect of serum E_2_ reduction on GC function. Our previously studies have demonstrated that GnRH agonist administration may lower, but not eliminate serum E_2_ levels in normal and PCOS women, particularly those with increased BMI as was the case with our study subjects [12]. To maintain reduced levels of E_2_ following ovarian suppression LET was employed during the interval of ovarian recovery. Use of an anti-estrogen such as a selective E_2_ receptor modulator may have been considered, although these compounds may bind to E_2_ receptors and activate signaling pathways independent of E_2_. In addition, the degree to which E_2_ action was reduced would be unclear and measurement of E_2_ levels would be of questionable value.

Clinical studies to examine the role of estrogen on GC function in PCOS or normal women have not been performed previously. However, the interpretation of our results warrants some caution due to the following limitations. First, there were a limited number of subjects that served as their own controls. Second, non-PCOS women were not included. Third, the stimulatory dose of FSH was relatively high and GC responses may have been maximally stimulated that precluded differential Inh B production. Fourth, in light of ovarian suppression by GnRH agonist, LET administration may have been extended to allow a lengthier recovery prior to FSH stimulation. This is unlikely as our previous studies as well as the current results suggest that adequate steroidogenesis had been reestablished by the time of FSH testing. Lastly, we elected to employ Inh B as a marker of GC responsiveness to FSH as suggested by Dokras, et. al [11]. This assumption was based on the parallelism of Inh B and E_2_ responses to variable doses of iv FSH in both normal and PCOS women as previously reported [12].

## Conclusions

In summary, the results of this study have demonstrated that in women with PCOS Inh B responses to FSH are reduced in the presence of lowered circulating E_2_ levels. These finding provide indirect evidence that E_2_ may enhance follicle function in women with this disorder.

## Abbreviations

17-OHP: 17-hydroxyprogesterone
A: Androstenedione
CTRI: Clinical and translational research institute
CV: Coefficients of variation
DHEAS: Dehydroepiandrosterone sulfate
E_2_: Estradiol
GCs: Granulosa cells
Inhibin B: Inh B
LET: Letrozole
PCOS: Polycystic ovary syndrome
r-hFSH: Recombinant human FSH
RIA: Radio-immunoassay
T: Testosterone
